# Development and characterisation of novel durum wheat*–H. chilense* 4H^ch^ chromosome lines as a source for resistance to Septoria tritici blotch

**DOI:** 10.3389/fpls.2024.1393796

**Published:** 2024-07-23

**Authors:** Zuny Cifuentes, Maria-Carmen Calderón, Cristina Miguel-Rojas, Josefina C. Sillero, Pilar Prieto

**Affiliations:** ^1^ Plant Breeding Department, Institute for Sustainable Agriculture, Agencia Estatal Consejo Superior de Investigaciones Científicas (CSIC), Córdoba, Spain; ^2^ Area of Plant Breeding and Biotechnology, IFAPA Alameda del Obispo, Córdoba, Spain

**Keywords:** Septoria leaf blotch, plant breeding, Ph1 locus, foliar disease, chromosome manipulation

## Abstract

The use of wild species as a source of genetic variability is a valued tool in the framework of crop breeding. *Hordeum chilense* Roem. *et* Schult is a wild barley species that can be a useful genetic donor for sustainable wheat breeding which carries genes conferring resistance to some diseases or increasing grain quality, among others. Septoria tritici blotch (STB), caused by the *Zymoseptoria tritici* fungus, is one of the most important wheat diseases worldwide, affecting both bread and durum wheat and having a high economic impact. Resistance to STB has been previously described in *H. chilense* chromosome 4H^ch^. In this study, we have developed introgression lines for *H. chilense* chromosome 4H^ch^ in durum wheat using interspecific crosses, advanced backcrosses, and consecutive selfing strategies. Alien *H. chilense* chromosome segments have been reduced in size by genetic crosses between *H. chilense* disomic substitution lines in durum wheat and durum wheat lines carrying the *Ph1* deletion. *Hordeum chilense* genetic introgressions were identified in the wheat background through several plant generations by fluorescence *in situ* hybridisation (FISH) and simple sequence repeat (SSR) markers. An STB infection analysis has also been developed to assess STB resistance to a specific *H. chilense* chromosome region. The development of these *H. chilense* introgression lines with moderate to high resistance to STB represents an important advance in the framework of durum breeding and can be a valuable tool for plant breeders.

## Introduction

Increasing human population and climate change demand greater agricultural productivity. Wheat is a strategic crop, essential for Western countries. It is the most important source of protein, providing 20% of the calories consumed daily by humans, and its consumption is increasing, mainly in developing countries. Although the yield of primary crops, including wheat, in 2022 was double that of 2000, it is estimated that the global demand for wheat production will increase by 60% in 2050 to meet the requirements of a growing population (http://www.fao.org). Europe is the first-world producer of bread wheat and within the European Union; Spain ranks in the fifth position considering cereal production, mainly wheat, only behind France, Germany, Romania, and Poland (http://www.fao.org). Thus, the economic relevance of wheat as a strategic crop in agriculture is clear, but several biotic (diseases) and abiotic (higher temperatures, soil erosion, salinity, water availability, etc.) stresses can compromise the future of this crop. A solid strategy, based on the development of new approaches that allow the adaptation of wheat to climate change and are respectful of the principles of sustainable agriculture, is needed to face the challenge of feeding the expected human population (Hamblin, 1995). Plant breeders are playing a major role in worldwide efforts to understand gene functions and interactions, so the introduction of tolerant genes to both biotic and abiotic stresses can increase the quality and productivity of a key crop such as wheat (Mamrutha et al., 2019; Mondal et al., 2021; Pérez-Méndez et al., 2022).

Durum wheat (*Triticum turgidum*, 2n = 4x = 28) is a globally relevant species for agriculture and also has an enormous impact in Spain since it is one of the most cultivated cereals together with bread wheat and barley. Despite its high productivity, annual fluctuations can be associated with biotic and abiotic stresses. One of the most important diseases affecting durum wheat is Septoria tritici blotch (STB) caused by the fungus *Zymoseptoria tritici* (Desm.) (Quaedvlieg et al., 2011), previously known as *Septoria tritici* (teleomorph *Mycospaherella graminicola* (Fuckel) J. Schröt.), which triggers severe losses in yield that can reach 50% in susceptible cultivars (Fones and Gurr, 2015). Severe epidemics occur both in areas with frequent rains and in others where rainfall does not exceed 250 mm during the crop cycle. The wide range of temperatures in which the fungus can develop, the use of susceptible varieties, and the rapid evolution and adaptation of *Z. tritici* to diverse agricultural conditions have allowed a wide geographical distribution of the disease (Fones and Gurr, 2015).

The introgression of resistance genes in the framework of breeding programs is considered the main protection against this disease, and several sources of resistance have yielded adequate protection (Rubiales et al., 2001). Wheat resistance to STB can be qualitative (isolate-specific), depending on major genes with a strong effect according to a gene-for-gene interaction, or quantitative (isolate-non-specific) displaying a partial phenotype controlled by various genes with moderate to small effects (Kema et al., 2000; Brading et al., 2002; Brown et al., 2015). Quantitative resistance is quite significant for its effectiveness against all genotypes of the pathogen and its durability (Brown et al., 2015; Porras et al., 2021). When no sufficient genetic variability or resistant varieties are available, the incorporation of new alleles into the wheat germplasm is considered essential. Thus, genetic crosses between donor species and the crop itself can be a powerful tool to transfer chromosome segments carrying desirable genes from the alien species into wheat.

Breeders have been using related species as genetic donors with the target of widening the genetic basis of this crop obtaining, for example, wheat cultivars better adapted to specific agro-climatic conditions, carrying resistance to pests (Lukaszewski, 2000; Shubing and Honggang, 2005). Indeed, wide-crossing in plant breeding programmes is such an important tool that, sometimes the results are the starting point for new crops (O’mara, 1953; Martin and Sanchez-Mongelaguna, 1982) and marked a milestone in a breeding framework.

Diploid wild barley *Hordeum chilense* Roem. *et* Schultz (2n = 2x = 14 H^ch^H^ch^) exhibits valuable agronomic and quality traits for wheat improvement, and it has already been crossed with some species of the tribe Triticeae (grasses), particularly with both durum and bread wheat (Martin et al., 1996; Calderón et al., 2012). This wild species also has enormous potential as a donor of other genetic features such as drought and salt tolerance as well as resistance to pests and diseases (Gallardo and Fereres, 1989; Bothmer et al., 1995; Martin et al., 1996). Particularly, *H. chilense* contains interesting genes for biotic and abiotic stress resistance, such as resistance to STB, conferred mainly by the 4H^ch^ chromosome (Rubiales et al., 2000). The transfer of *H. chilense* chromosome 4 in durum wheat has been already developed (Calderón et al., 2012), but, in a plant breeding framework, the size of full introgressed chromosomes from relatives must be reduced to diminish the drag linkage of undesirable genes.

Much of the work aimed to promote interspecific chromosome associations to transfer genetic variability from wild relatives into wheat has relied on the use of the *ph1b* mutant (Sears and Okamoto, 1958). Homoeologous recombination between chromosomes from related but different genomes can occur in the absence of the *Ph1* locus, resulting in the generation of interspecific recombinant chromosomes (Sears and Okamoto, 1958; Othmeni et al., 2019). In the absence of the *Ph1* locus, chromosome rearrangements in bread wheat involving wheat and relative *H. chilense* and *H. vulgare* chromosomes have already been obtained (Rey et al., 2015a, 2015). In this work, we used the *ph1* mutant and the previously developed *H. chilense* chromosome 4H^ch^ substitution line (Calderón et al., 2012), both in durum wheat background, to carry out genetic crosses to facilitate durum wheat-wild barley interspecific recombination. A set of durum wheat–*H. chilense* chromosome 4H^ch^ translocation lines were obtained and characterised using molecular markers and fluorescence *in situ* hybridisation. We also analysed these lines for the incidence of STB under controlled conditions in growth chambers to assess resistance to STB to an *H. chilense* chromosome 4H^ch^ segment.

## Materials and methods

### Plant materials

**Figure 1:**
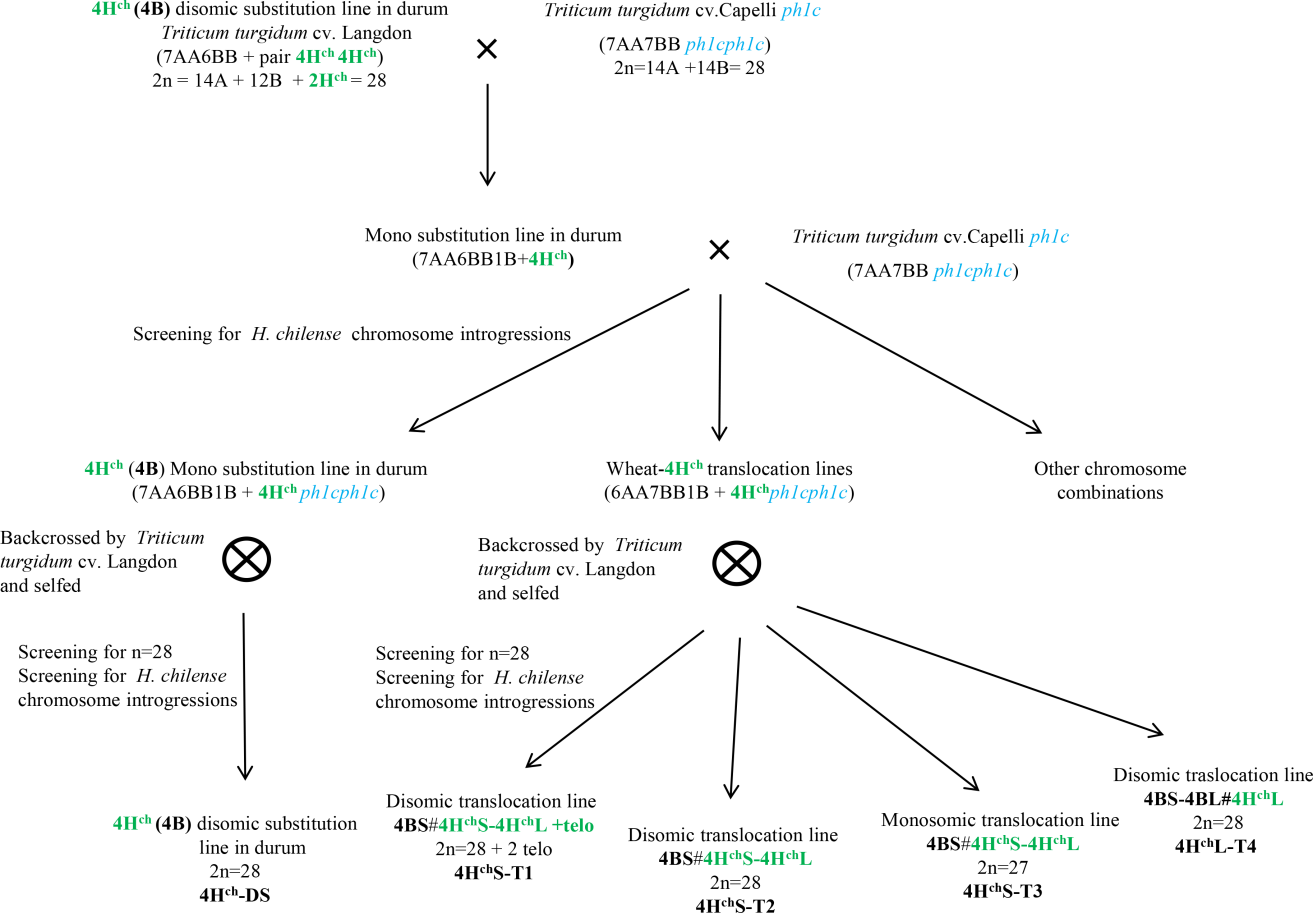
Development of *H. chilense* introgression lines in tetraploid durum wheat (*Triticum turgidum* cv. Langdon). Crosses between the 4H^ch^ substitution line in *T. turgidum* and the *ph1c* wheat mutant (*Triticum turgidum* cv. Capelli) were developed to obtain *H. chilense* genetic introgressions in the absence of the *Ph1* locus. Backcrosses by durum wheat (*Triticum turgidum* cv. Langdon) were developed. Screening for *H. chilense* genetic introgressions was carried out using molecular markers and *in situ* hybridization.

Genetic crosses between the 4H^ch^ (4B) substitution line in durum wheat cv. Langdon (*T. turgidum*, 2n=4x = 28; (Calderón et al., 2012) and durum wheat cv. Capelli carrying the *Ph1* deletion (Ceoloni et al., 1992) have been carried out. F1 plants were backcrossed with durum wheat cv. Capelli in the *ph1* mutant background. Plants were selected using molecular markers and by *in situ* hybridisation and then selfed. The procedure is described in **Figure 1**, and the chromosomal composition of *Hordeum chilense* chromosome 4H^ch^ introgression lines in durum wheat is summarized in **Table 1**. Plants carrying *H. chilense* introgressions were selected for analysis.

**Table 1:** Progeny of the crosses between the 4H^ch^(4B) substitution line in durum wheat (*Triticum turgidum*) and the durum wheat carrying the *Ph1* deletion.

Plant line	Chromosome number	Chromosomal composition	Number of plants obtained (% fertility)
4H^ch^-DS	28	4H^ch^ disomic substitution line	103 (100%)
4H^ch^S-T1	28 + 2 telo	Disomic translocation 4BS#4H^ch^S-4H^ch^L+telo	45 (50%)
4H^ch^S-T2	28	Disomic translocation 4BS#4H^ch^S-4H^ch^L	29 (50%)
4H^ch^S-T3	27	Monosomic translocation 4BS#4H^ch^S-4H^ch^L	59 (50%)
4H^ch^L-T4	28	Disomic translocation 4BS-4BL#4H^ch^L	52 (40%)

Chromosomal composition of *Hordeum chilense* chromosome 4H^ch^ introgression lines in durum wheat after backcrossed and selfed generations.

Seeds were incubated in Petri dishes on wet filter paper in the dark for 5 days at 4°C, followed by 24 h of incubation at 24°C to allow germination. Then, roots were cut and incubated for 4 h in 0.05% colchicine solutions at 24°C to accumulate somatic cells in metaphase and finally fixed in ethanol/glacial acetic acid 3:1 (v/v) for 1 month at 4°C.

### Cytogenetic analysis

For identification and characterisation of the *H. chilense* chromosome 4H^ch^ introgressions and wheat chromosomes involved in translocations, fluorescence *in situ* hybridisation (FISH) using *H. chilense* DNA, the GAA-satellite sequence, and the pAs1 probe (Cabrera et al., 1995; Pedersen et al., 1997) was performed. DNA probes were labelled by nick translation with biotin-11 dUTP (Roche Corporate, Basel, Switzerland) and digoxigenin 11-dUTP (Roche Corporate, Basel, Switzerland) and detected with streptavidin-Cy3 (Sigma, St Louis, MO, USA) and anti-digoxigenin–FITC (Roche Corporate Basel, Switzerland), respectively.

Metaphase mitotic chromosomes were hybridised with total genomic *H. chilense* DNA as a probe to identify the chromosome segment introgressed from *H. chilense* chromosome 4H^ch^. Afterwards, the preparations were hybridised using the repetitive sequence GAA and the pAs1 probe to characterise both the *H. chilense* chromosome segment and the wheat chromosome involved in interspecific chromosome translocations. The *in situ* protocol was performed according to Prieto et al. (2004).

Hybridisation signals were visualised using a Nikon eclipse inflorescence microscopy, and the images were captured with a Nikon CCD camera using the appropriate Nikon 3.0 software (Nikon Instrument Europe BV, Amstelveen, The Netherlands) and processed with Photoshop 4.0 software for brightness and contrast (Adobe Systems Inc., San Jose, CA, USA).

### Molecular marker characterisation

Molecular markers were selected and tested for the specific identification of the short and long arms of *H. chilense* chromosome 4H^ch^ according to available information (Said and Cabrera, 2009; Sakata et al., 2010).

Genomic DNA was extracted from frozen young leaves using the CTAB method described by Murray and Thompson (1980). We tested 25 and 9 markers to specifically identify both the short arm and the long arm of chromosome 4H^ch^, respectively. The polymerase chain reaction (PCR) experiments were performed in 20 µL of a mixture containing 4 µL of reaction buffer containing MgCl_2_ and dNTPs, 5 pmol of selected primers, and 0.4 µL of MyTaq DNA polymerase. Specific primers and PCR conditions to amplify these markers were according to Masaya Sakata et al. (2010) and Said and Cabrera (2009). The PCR products were visualized using SafeView (NBS Biologicals, Cambridgeshire, UK) in an agarose (1%) electrophoresis gel (120 V, 45 min).

We have also used the NCBI’s BLAST (Basic Local Alignment Search Tool) to perform retro-transcription-PCR (RT-PCR) *in silico* on *H. chilense* genome and to locate the position of the primers on the *H. chilense* physical map using the reference genome (txid15565) from the NCBI database (National Center for Biotechnology Information).

### Infection assay

The inoculation process was developed as reported by Stewart and McDonald (2014), following minor modifications described by Porras et al. (2021). Briefly, seeds of the 10 durum wheat genotypes, including the positive control, were sown in 8 × 7 × 7 cm pots containing a mix (1:1, *v*/*v*) of commercial compost (Suliflor SF1 substrate; Suliflor Lithuania) and sand. Pots were incubated in a growth chamber at 21°C and 70% relative humidity (RH), with 16 h of light. After 16 days, seedlings were fungal inoculated when the second and third leaves emerged [growth stage Z13; (Zadoks et al., 1974)]. *Zymoseptoria tritici* (isolated from infected durum wheat of Santaella (Cordoba, Spain) and deposited at the NBCI database under the SUB9540116 accession number) inoculum was adjusted to 10^7^ spores mL^−1^ in distilled water and 0.1% Tween 20. Seedlings were inoculated using a hand sprayer. Once leaves were totally dry, plants were sealed in clear plastic bags to provide 100% RH and maintained for 48 h in a growth chamber at 22/18°C day/night with a 16-h photoperiod. After 48 h, plastic bags were removed, and plants were kept at 75%–80% RH. The amount of infected plants per family was variable, ranging from 6 to 18, based on seed availability. The positive control check, the commercial cultivar ‘SY Leonardo’, was inoculated following the same treatment. Three independent experiments with three replicates each were performed.

### Disease assessment

At 21 days post-inoculation, the third leaf of each plant was evaluated. The infection process was qualitatively scored using the disease severity (DS) rating scale from 0 to 5 (McCartney et al., 2002), as follows: 0 = immune with no visible symptoms, 1 = highly resistant with hypersensitive flecking, 2 = resistant with small chlorotic or necrotic lesions and no pycnidial development, 3 = moderately resistant, characterised by coalescence of chlorotic and necrotic lesions with slight pycnidial development, 4 = susceptible with moderate pycnidial development and coalesced necrotic lesions, and 5 = very susceptible with large, abundant pycnidia and extensively coalesced necrotic lesions. Porras et al. (2021) showed a digitally infected leaves scale with STB that was used as a reference to evaluate the families of the present study. Reaction types 0–3 were considered resistant, whereas reaction types 4 and 5 were considered susceptible. Although reaction type 3 includes pycnidium development, it is considered resistant because the growth and sporulation of the fungus are quite restricted, and the chlorotic reaction is similar to the chlorotic blotches of reaction type 2.

## Results

### Cytogenetic and molecular marker analysis

Crosses between the 4H^ch^(4B) substitution line in durum wheat cv. Langdon and the durum wheat cv. Capelli carrying the *Ph1* deletion were carried out to promote interspecific recombination between chromosome 4H^ch^ and the homoeologous wheat chromosomes in the absence of the *Ph1* locus. To obtain the *Ph1* deletion in homozygosis, one backcross to durum wheat cv. Capelli carrying the *Ph1* deletion was carried out. Wheat plants carrying monosomic and disomic *H. chilense* introgressions were selected and analysed by multicolour *in situ* hybridisation and molecular markers. The procedure is summarised in **Figure 1**. We obtained more than 200 plants having either monosomic or disomic genetic introgressions involving *H. chilense* chromosome 4H^ch^ in durum wheat (**Table 1**).

A total of 24 EST-SSR (Expressed Sequence Tags-Simple Single Repeat) specific markers for the short arm of *H. chilense* chromosome 4H^ch^ and nine barley markers for the long arm of the same chromosome were tested to identify those markers displaying polymorphism between durum wheat and the wild barley (**Table 2**). Most of them did not show polymorphism between the 4H^ch^ *H. chilense* and the 4A wheat homoeologous chromosome and were not used for the genetic screening. Only 10 markers revealed polymorphism between 4H^ch^ *H. chilense* and wheat and were used to identify and characterise the size of the 4H^ch^ *H. chilense* introgressions (**Table 3**).

**Table 2:** Molecular markers used for the screening of *Hordeum chilense* chromosome introgressions in the durum wheat (DW) background.

Markers for 4H^ch^	DW	*H. chilense* (line H1)	4H^ch^-DS
4H^ch^S			
K07854	×	×	×
K03491	–	–	–
**K00607**	**-**	**x**	**x**
K00967	×	×	×
K03028	×	×	–
K00266	×	×	×
K00201	–	–	–
K02131	×	×	×
K00230	×	×	×
K02013	×	×	×
K01265	×	×	×
K04643	×	×	×
**BAWU505**	**-**	**x**	**x**
K02029	×	×	×
**GBM1028**	**-**	**x**	**x**
K00783	×	×	×
**BAWU303**	**-**	**x**	**x**
**K02529**	**-**	**x**	**x**
MWG542	–	×	–
K07933	×	×	×
K05013	×	×	×
K06310	×	×	×
K00552	×	×	×
BAWU852	–	×	–
4H^ch^L			
K05042	×	×	×
K04115	×	×	×
K00136	×	×	–
K03089	–	–	–
**BAWU217**	**-**	**x**	**x**
**BAWU152**	**-**	**x**	**x**
**BAWU312**	**-**	**x**	**x**
**BAWU497**	**-**	**x**	**x**
**K04754**	**-**	**x**	**x**

Polymorphic markers between durum wheat and *H. chilense* line H1 (in bold) were used for the assessment of the introgression lines. Although BAWU852 and MWG542 markers showed polymorphism between durum wheat and the H1 *H. chilense* line, signals were not strong enough to be used for a reliable screening and were discarded.

**Table 3:** Barley SSR markers used for the identification of *Hordeum chilense* chromosome 4H^ch^ introgressions in the durum wheat (DW) background.

Marker	Genetic position	Genotype						
		DW	H1	4H^ch^-DS	4H^ch^S-T1	4H^ch^S-T2	4H^ch^S-T3	4H^ch^L-T4
4H^ch^S								
K00607	27.6 cM (Sakata et al., 2010)	–	×	×	–	–	–	–
BAWU505	70% distal (Said and Cabrera, 2009)	–	×	×	–	–	–	–
GBM1028	52.3 cM (Said and Cabrera, 2009)	–	×	×	–	–	–	–
BAWU303	70% distal (Said and Cabrera, 2009)	–	×	×	×	×	–	–
K02539	106.0cM (Sakata et al., 2010)	–	×	×	×	×	×	–
4H^ch^L								
BAWU217	70% distal (Said and Cabrera, 2009)	–	×	×	×	×	×	–
BAWU152	70% distal (Said and Cabrera, 2009)	–	×	×	×	×	×	–
BAWU497	70% distal (Said and Cabrera, 2009)	–	×	×	×	×	×	–
BAWU312	70% distal (Said and Cabrera, 2009)	–	×	×	×	×	×	×
K04754	249.0 cM (Sakata et al., 2010)	–	×	×	×	×	×	×

All the durum wheat–*H. chilense* translocation lines obtained in this work were identified and characterised using this set of 10 markers.

The chromosomal location of polymorphic molecular markers on the 4H^ch^ chromosome was performed by *in silico* RT-PCR on the *H. chilense* genome, using NCBI’s BLAST for lining up the preexisting primers. Five markers were located on the short arm of chromosome 4H^ch^ (K00607, BAWU505, GBM1028, BAWU303, and K02539) and another five on the 4H^ch^L (BAWU217, BAWU152, BAWU497, BAWU312, and K04754). The molecular analysis of the four translocation lines obtained showed that marker K02539, located near the centromere of 4H^ch^S, amplified in 4H^ch^S-T1, 4H^ch^S-T2, and 4H^ch^S-T3 and was absent in 4H^ch^L-T4, revealing that wheat–*H. chilense* recombination in 4H^ch^S-T1, 4H^ch^S-T2, and 4H^ch^S-T3 did occur between 4H^ch^S and wheat chromosomes. Only 4H^ch^S-T1 and 4H^ch^S-T2 were positive for the BAWU303 marker. K00607, BAWU505, and GBM1028 markers, located in a distal position on 4H^ch^S, were absent in all translocation lines (**Table 3**). Thus, three wheat–*H. chilense* translocation lines involving the short arm of chromosome 4H^ch^ were obtained. Recombination events did occur at least in two different positions on the 4H^ch^ chromosome as 4H^ch^S-T1 and 4H^ch^S-T2 displayed a similar molecular pattern and were different from the 4H^ch^S-T3 translocation line. Related to the molecular analysis of those markers located on the long arm of 4H^ch^ *H. chilense* chromosome, all translocation lines were positive for distal BAWU312 and K04754 markers. BAWU217, BAWU152, and BAWU497 were positive in all translocation lines except in 4HchL-T4. In fact, the 4H^ch^L-T4 translocation line was only positive for the distal K04754 and BAWU312 markers. Our results displayed that this 4H^ch^L-T4 translocation line only retained a distal 4H^ch^L chromosome segment in the durum wheat background.

**Figure 2:**
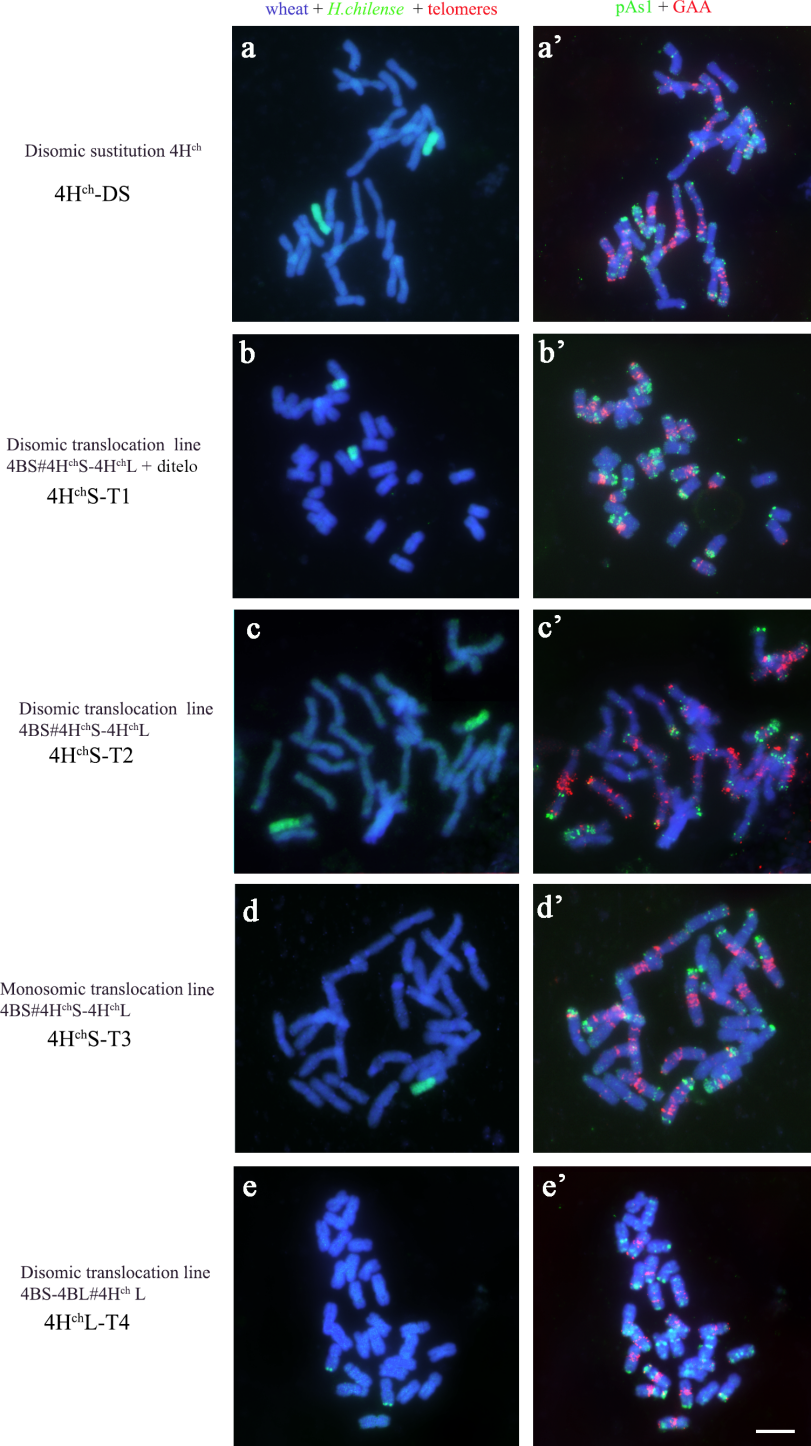
Cytogenetic visualisation of the introgression lines obtained for chromosome 4H^ch^ in *Triticum turgidum* using fluorescence *in situ* hybridisation*. Hordeum chilense* genomic introgressions are shown in green **(A-E)**. Chromosome identification and orientation were confirmed by reprobing with the pAs1 (green) and GAA (red) probes in **(A′-E′)**. DNA was counterstained with DAPI (blue). **(A-A′)** 4H^ch^ disomic substitution line; **(B-B′)** 4H^ch^S-T1 translocation line; **(C-C′)** 4H^ch^S-T2 translocation line, **(D-D′)** 4H^ch^S-T3 translocation line; **(E-E′)** 4H^ch^L-T4 translocation line. Scale bar for all panels = 10 µm.

**Figure 3:**
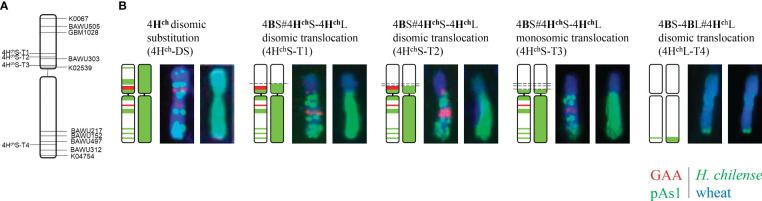
Ideogram of the wheat*-H. chilense* recombinant chromosomes. **(A)** Graphical genotyping of the chromosome indicating the recombination breakpoints of the SSR markers. **(B)** Characterisation of the *H. chilense* segment introgressed using the pAs1 (in green) and GAA (in red) probes. The whole 4H^ch^ chromosome segment is also shown (green).

Depending on the recombination event between *H. chilense* and wheat chromosomes, four different types of genetic introgressions for *H. chilense* chromosome 4H^ch^ were obtained in durum wheat in the *ph1* mutant background (**Table 1**; **Figures 2**, **3**). Most of the interspecific recombination events that we were able to recover occurred between the short 4H^ch^ and 4A chromosome arms. Thus, we obtained three different translocation lines involving the short 4H^ch^ and 4A chromosome arms, named 4H^ch^S-T1, 4H^ch^S-T2, and 4H^ch^S-T3 and one involving the long 4H^ch^ and 4A chromosome arms, named 4H^ch^L-T4.

Genomic *in situ* hybridisation contributed to elucidate the exact chromosomal composition of all translocation lines obtained (**Figure 2**). All the *H. chilense*–wheat translocation lines obtained were disomic except 4H^ch^S-T3, which was monosomic. The cytogenetic analysis using the psA1 and GAA repeat sequences contributed to characterise the chromosome segment of the *H. chilense* chromosome 4H^ch^ introgressed in durum wheat by their specific pattern on this chromosome 4H^ch^ (**Figure 3B**). Thus, 4H^ch^S-T1, 4H^ch^S-T2, and 4H^ch^S-T3 translocation lines contained the full 4H^ch^L chromosome arm and a fragment of the 4H^ch^S chromosome arm, which vary among these translocation lines depending on the position of the recombination event (**Figure 3B**). The cytogenetic analysis using the psA1 and GAA repeat sequences revealed a similar cytogenetic pattern between 4H^ch^S-T1 and 4H^ch^S-T2 translocation lines (**Figure 3B**), which agrees with the equivalent result obtained using molecular markers (**Table 3**). These results suggest that the recombination events occurring between wheat and barley chromosomes in these two 4H^ch^S-T1 and 4H^ch^S-T2 translocation lines were extremely close to each other along the 4H^ch^S chromosome. In addition, a recombination event on the distal region of chromosome 4H^ch^L did occur in the 4H^ch^L-T4 translocation line, retaining only a small 4H^ch^L chromosome segment (**Figures 2**, **3**).

### STB infection analysis

**Figure 4:**
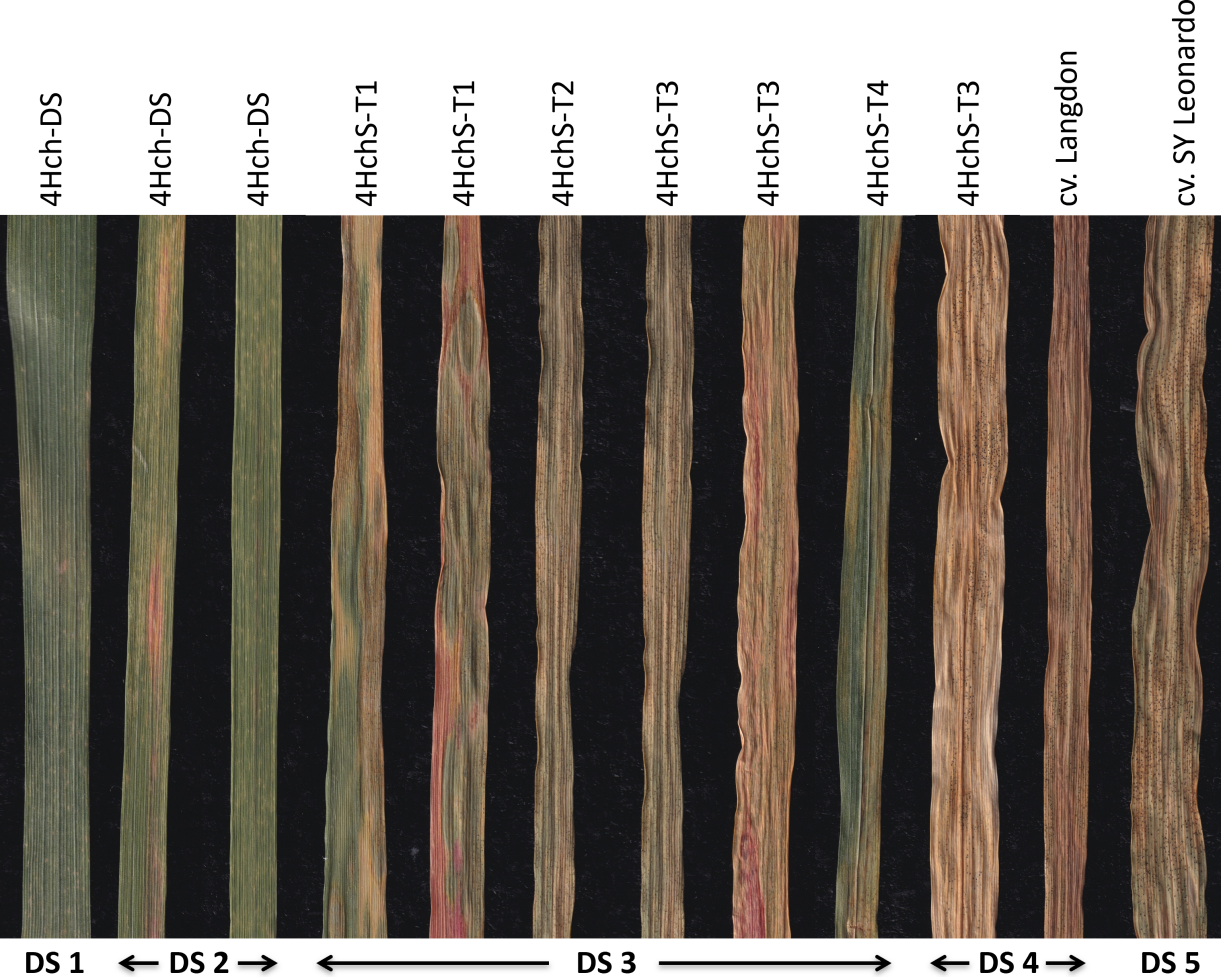
Examples of infected leaves with Septoria tritici blotch (STB) showing diverse disease severity rating scale (DS) scores. Leaves from the studied genotypes with different average DS scores. The 4H^ch^ disomic substitution, 4H^ch^-DS, line displayed DS1-2. All the translocation lines 4H^ch^S-T1, 4H^ch^S-T2, 4H^ch^S-T3, and 4H^ch^L-T4 displayed DS 3, although some leaves from 4H^ch^S-T3 also showed DS4 as the durum wheat cultivar Langdon. The most sensitive line (DS5) was the control cultivar SY Leonardo.

The development of STB in the *H. chilense* translocation lines obtained in the durum wheat background is shown in **Table 4** and **Figure 4**. Two different experiments were carried out, and differences in Disease Severity (DS) rating scale scores were recorded. DS scores showed differences among the plants studied, ranging from 2 to 4. Most of the studied durum wheat lines exhibited a resistant to moderately resistant reaction, displaying DS ≤ of 3, which indicated a restricted growth and sporulation of the fungus. The 4H^ch^ *H. chilense* disomic substitution line (4H^ch^-DS) could be considered the most resistant one, showing a heterogeneous behaviour against the disease but with the lowest DS scores (1-2). This means that some leaves presented reaction types highly resistant with hypersensitive flecking (DS 1), and others showed small necrotic lesions with no pycnidia development (DS 2). No remarkable differences were found between durum wheat–*H. chilense* translocation lines 4H^ch^S-T1, 4H^ch^S-T2, and 4H^ch^L-T4, showing an average DS score of 3 (moderately resistant), with limited production of pycnidia (DS 3) and small necrosis lesions. In lines 4H^ch^S-T2 and 4H^ch^L-T4, most leaves displayed a mainly moderately resistant response with limited production of pycnidia with chlorotic/necrotic lesions (DS 3), although some leaves allowed higher pycnidia development. Only the family 4H^ch^S-T3 displayed an average DS score higher than 3, showing leaves with two different reaction types, moderately resistant (DS 3) and susceptible (DS 4). This result could be because this translocation line has only one copy of the *H. chilense* chromosome segment (monosomic translocation line). Lastly, durum wheat cv. Langdon, which is the genetic background of the translocation lines developed in this work, presented an average DS score of 4 (susceptible), which indicates significant fungus-reproduction capability in the form of pycnidia in the necrotic lesions. Strikingly, none of the analysed durum wheat including the Langdon cultivar showed a DS score of 5 (very susceptible with abundant pycnidia and extensively coalesced necrotic lesions). As it was expected, the other control used in this work, cultivar SY Leonardo, was very susceptible, showing most of the screened plants high reproduction of the fungus and extensively coalesced lesions (DS 5).

**Table 4:** Classification of studied families according to their Disease Severity (DS) rating scale (McCartney et al., 2002).

Line	Number of plants	Percentage of plants with DS score					Average	Reaction types
		DS 1	DS 2	DS 3	DS 4	DS 5		
4H^ch^-DS	27	36	59	5	0	0	1-2	R
4H^ch^S-T1	18	0	22	67	17	5	3	MR
4H^ch^S-T2	6	0	0	67	33	0	3	MR
4H^ch^S-T3	24	0	4	57	39	0	3-4	MS
4H^ch^L-T4	9	0	0	78	22	0	3	MR
Langdon	14	0	0	21	79	0	4	S
SY Leonardo	10	0	0	0	20	80	5	S

The commercial cultivar ‘SY Leonardo’ and durum wheat cv. Langdon were used as positive control of STB infection. R, resistant; MR, moderately resistant; MS, moderately susceptible; S, susceptible.

DS rating scale: 1 = highly resistant with hypersensitive flecking, 2 = resistant with small chlorotic or necrotic lesions and no pycnidial development, 3 = moderately resistant, characterised by coalescence of chlorotic and necrotic lesions with slight pycnidial development, 4 = susceptible with moderate pycnidial development and coalesced necrotic lesions, and 5 = very susceptible with large, abundant pycnidia and extensively coalesced necrotic lesions.

## Discussion

The effective introgression of genes from alien species like *H. chilense* into cultivated species such as durum wheat entails that target genes or the chromosome segment carrying them must be incorporated into wheat chromosomes as recombinant segments or translocations. Examples of an important source of chromosome introgressions are centric-break fusion events found several decades ago in bread wheat–rye translocations (e.g., 1BL-lRS and lAL-lRS), which have importantly contributed to global wheat production (Pena et al., 1990). Genetic introgressions from *H. chilense* chromosome 4H^ch^, which contains resistance to STB (Rubiales et al., 2000), have been previously developed in the durum wheat background (Calderón et al., 2012). In this work, genetic crosses between the durum wheat line carrying a disomic 4H^ch^(4B) chromosome substitution and durum wheat carrying the *Ph1* deletion were developed to promote interspecific recombination between wheat and *H. chilense*. Recombination events between bread wheat and *H. chilense* lines in the absence of the *Ph1* locus have been previously promoted for both fundamental and applied purposes, which is to shed light into the mechanism of this *Ph1* locus or in a plant breeding framework to specifically introgress *H. chilense* chromosome 7H^ch^ to increase the carotenoid content in bread wheat (Rey et al., 2015a). To the best of our knowledge, it is the first time that the *ph1* mutant has been used in genetic crosses between *H. chilense* and wheat in the tetraploid durum background. The use of the *ph1* mutants in this work has enabled the possibility of promoting recombination events between 4A wheat and 4H^ch^ *H. chilense* homoeologous chromosomes, providing the opportunity to introgress part of 4H^ch^ chromosome in durum wheat and the development of a series of genetic introgressions for this *H. chilense* chromosome for durum breeding purposes. The durum wheat–*H. chilense* introgression lines for chromosome 4H^ch^ developed in this work might facilitate the possibility of transferring resistance to STB in durum wheat as the 4H^ch^ chromosome was previously targeted as a potential source to introduce this type of resistance in wheat (Rubiales et al., 2000).

The interaction between *Z. tritici* and durum wheat has been poorly studied, and no *Stb* genes have been identified in durum wheat so far (Somai-Jemmali et al., 2017). This lack of identified and characterised *Stb* resistance genes is a challenge for plant breeders to find durable sources of resistance to STB in durum wheat. Resistance to STB is generally expressed through the reduction of the foliar area covered with pycnidia and necrosis (Porras et al., 2021). In this work, we have obtained several durum wheat–*H. chilense* translocation lines including chromosome 4H^ch^ that present a moderate resistance to STB. The reduction of the diseased in the 4H^ch^ *H. chilense* introgression lines in durum wheat is attributed to the contribution of chromosome 4H^ch^ compared with both control lines, the cultivar Langdon, which is the genetic background of the *H. chilense* introgressions developed, and the cultivar SY Leonardo, which is routinely used as a susceptible control to STB. Both durum cultivars displayed elevated susceptibility with abundant development of the fungus and extensively coalesced lesions (DS 4 and 5, respectively) in contrast to the DS scores 1 and 2 expressed by the 4H^ch^ disomic substitution line, 4h^ch^-DS, or the average DS score of 3 displayed by the translocation lines 4H^ch^S-T1, 4H^ch^S-T2, and 4H^ch^L-T4. Thus, our results demonstrate the contribution of chromosome 4H^ch^ for introgressing resistance to STB in durum wheat.

The combination of cytogenetic tools with a molecular analysis offers a reliable method of selection of *H. chilense* introgressions in the durum wheat background during a plant breeding programme. These combined approaches provide the possibility to detect and analyse a high number of plant lines carrying chromosome modifications in a suitable frame of time, and for this, they have been extensively used for the identification and characterisation of plant material for decades (Nalam et al., 2006; Faris et al., 2008; Wang et al., 2009; Calderón et al., 2012). In this work, genomic *in situ* hybridisation contributed to elucidate the exact chromosomal composition of all translocation lines obtained. All the *H. chilense*–wheat translocations obtained were disomic except 4H^ch^S-T3, which was monosomic. Disomic 4H^ch^S-T1, 4H^ch^S-T2, and 4H^ch^L-T4 translocation lines showed moderate resistance to STB, with limited production of pycnidia and small necrosis lesions, although the monosomic 4H^ch^S-T3 line displayed moderately susceptible reaction with limited pycnidia development (DS 3-4). In contrast, the disomic line 4H^ch^-DS showed a resistant behaviour to STB displaying hypersensitive flecking (DS1) and necrotic lesions with no presence of pycnidia (DS 2). These results confirmed an apparent dosage effect on STB resistance as it was previously described in *Agropyron*–wheat derivates (Rillo et al., 1970) and agree with the presence of different *Stb* genes along the *H. chilense* chromosome 4H^ch^.

The similar pattern obtained by the cytogenetic analysis and molecular markers in 4H^ch^S-T1 and 4H^ch^S-T2 translocation lines suggested that the recombination events occurring in each of these translocation lines were extremely close to each other along the 4H^ch^S chromosome. The fact of being the same recombination event is discarded as both 4H^ch^S-T1 and 4H^ch^S-T2 translocation lines were obtained from different genetic crosses between the original 4H^ch^disomic line and the *ph1* mutants.

All the durum wheat–*H. chilense* translocation lines obtained in this work retained the distal region of *H. chilense* chromosome 4H^ch^L, which might contain some homology to bread wheat chromosome 4AL, where QTL7 and *Stb7* and *Stb12* resistance genes are located (Brown et al., 2015). All the durum wheat–*H. chilense* translocation lines developed in this work (4H^ch^S-T1, 4H^ch^S-T2, 4H^ch^S-T3, and 4H^ch^L-T4) displayed moderate resistance to STB compared with the control lines. However, some other gene(s) might also be located on the distal region of chromosome 4H^ch^S, as the 4H^ch^ *H. chilense* disomic substitution (4H^ch^DS) line could be considered the most resistant one, showing a heterogeneous behaviour against the disease but with the lowest DS scores (1–2). In addition, no remarkable differences in the STB resistance were found between 4H^ch^S-T1 and 4H^ch^S-T2 translocation lines, indicating that putative resistance genes might not be located in the small segment of chromosome 4H^ch^S in which these two durum wheat–*H. chilense* translocation lines might be different. Thus, that putative additional gene(s) should be included in the most distal 4H^ch^S chromosome region. Consequently, our results suggest that *H. chilense* chromosome 4H^ch^L might contain genes conferring resistance to STB on the distal 4H^ch^L chromosome arm. These genes could be homoeologous to those *Stb7* and *Stb12* resistance genes or STB7 QTL described previously in the 4A bread wheat chromosome (Brown et al., 2015), but, interestingly enough, additional gene(s) conferring stronger resistance to STB might also be included in the distal region of the *H. chilense* chromosome 4H^ch^S. To sum up, this present study sheds light on the interaction of *Z. tritici* and durum wheat, giving some clues to identify possible *Stb* genes in durum wheat. Therefore, the plant material obtained in this work constitutes a valuable germplasm to introduce resistance to STB in durum wheat.

## Data availability statement

The original contributions presented in the study are included in the article/supplementary material, further inquiries can be directed to the corresponding authors. *Zymoseptoria tritici* isolated is deposited in the public NBCI database under the MZ026796 accession number.

## Author contributions

ZC: Writing – original draft, Investigation. M-CC: Investigation, Writing – review & editing. CM-R: Investigation, Writing – review & editing. JS: Investigation, Writing – review & editing. PP: Conceptualization, Funding acquisition, Investigation, Project administration, Resources, Supervision, Writing – original draft, Writing – review & editing.
